# Initiating guideline-concordant gout treatment improves arterial endothelial function and reduces intercritical inflammation: a prospective observational study

**DOI:** 10.1186/s13075-020-02260-6

**Published:** 2020-07-11

**Authors:** Michael Toprover, Binita Shah, Cheongeun Oh, Talia F. Igel, Aaron Garza Romero, Virginia C. Pike, Fatmira Curovic, Daisy Bang, Deana Lazaro, Svetlana Krasnokutsky, Stuart D. Katz, Michael H. Pillinger

**Affiliations:** 1 Section of Rheumatology, VA New York Harbor Health Care System, New York, NY USA; 2 Division of Rheumatology, NYU Grossman School of Medicine, NYU Hospital for Joint Diseases Suite 1410, 301 East 17th Street, New York, NY 10003 USA; 3 Section of Cardiology, VA New York Harbor Health Care System, New York, NY USA; 4 Division of Cardiology, Department of Medicine, NYU Grossman School of Medicine, New York, NY USA; 5 Division of Biostatistics, Department of Population Health, NYU Grossman School of Medicine, New York, NY USA

**Keywords:** Gout, Hyperuricemia, Subclinical cardiovascular disease, Inflammation, Urate-lowering therapy, Colchicine, Xanthine oxidase inhibitor, Allopurinol, Flow-mediated dilation, C-reactive protein

## Abstract

**Background:**

Patients with gout have arterial dysfunction and systemic inflammation, even during intercritical episodes, which may be markers of future adverse cardiovascular outcomes. We conducted a prospective observational study to assess whether initiating guideline-concordant gout therapy with colchicine and a urate-lowering xanthine oxidase inhibitor (XOI) improves arterial function and reduces inflammation.

**Methods:**

Thirty-eight untreated gout patients meeting American College of Rheumatology (ACR)/European League Against Rheumatism classification criteria for gout and ACR guidelines for initiating urate-lowering therapy (ULT) received colchicine (0.6 mg twice daily, or once daily for tolerance) and an XOI (allopurinol or febuxostat) titrated to ACR guideline-defined serum urate (sU) target. Treatment was begun during intercritical periods. The initiation of colchicine and XOI was staggered to permit assessment of a potential independent effect of colchicine. Brachial artery flow-mediated dilation (FMD) and nitrate-mediated dilation (NMD) assessed endothelium-dependent and endothelium-independent (smooth muscle) arterial responsiveness, respectively. High-sensitivity C-reactive protein (hsCRP), IL-1β, IL-6, myeloperoxidase (MPO) concentrations, and erythrocyte sedimentation rate (ESR) assessed systemic inflammation.

**Results:**

Four weeks after achieving target sU concentration on colchicine plus an XOI, FMD was significantly improved (58% increase, *p* = 0.03). hsCRP, ESR, IL-1β, and IL-6 also all significantly improved (30%, 27%, 19.5%, and 18.8% decrease respectively; all *p* ≤ 0.03). Prior to addition of XOI, treatment with colchicine alone resulted in smaller numerical improvements in FMD, hsCRP, and ESR (20.7%, 8.9%, 13% reductions, respectively; all non-significant), but not IL-1β or IL-6. MPO and NMD did not change with therapy. We observed a moderate inverse correlation between hsCRP concentration and FMD responsiveness (*R* = − 0.41, *p* = 0.01). Subgroup analyses demonstrated improvement in FMD after achieving target sU concentration in patients without but not with established cardiovascular risk factors and comorbidities, particularly hypertension and hyperlipidemia.

**Conclusions:**

Initiating guideline-concordant gout treatment reduces intercritical systemic inflammation and improves endothelial-dependent arterial function, particularly in patients without established cardiovascular comorbidities.

## Background

Gout is the most common crystal arthropathy and the leading cause of inflammatory arthritis [1]. The past several decades have seen a surge in gout prevalence, along with associated increases in functional impairment and diminished health-related quality of life [2]. Epidemiological evidence suggests an association between gout and cardiovascular disease [3, 4]. In addition to increased rates of traditional cardiovascular risk factors in patients with gout [5, 6], gout conveys an independent risk for adverse cardiovascular outcomes [7, 8]. The fundamental pathological processes associated with gout, hyperuricemia, and local and systemic inflammation are independently implicated in the development and progression of cardiovascular disease [9–11] and may contribute to the increased cardiovascular risk observed in gout patients.

Management guidelines from the American College of Rheumatology (ACR) and the European League Against Rheumatism (EULAR) emphasize that most gout patients should be treated with a urate-lowering therapy (ULT), ideally a xanthine oxidase inhibitor (XOI), to a serum urate (sU) of ≤ 6.0 mg/dL, or potentially ≤ 5.0 mg/dL in the case of patients with tophaceous gout [12–14]. Because urate lowering transiently raises the risk of gout flares, gout treatment guidelines additionally recommend daily administration of an anti-inflammatory agent such as colchicine in the first months of ULT [13–15]. Whether management of gout per current guidelines reduces the risk of adverse cardiovascular outcomes is an area of ongoing investigation. Several studies suggest that colchicine use may be associated with a reduction in cardiac events both in gout patients [16, 17] and in non-gout patients at high cardiovascular risk [18, 19]. However, these studies were either retrospective or open-label or, in the case of the randomized Colchicine Cardiovascular Outcomes (COLCOT) trial, showed benefit largely through reductions in the cardiovascular endpoint of urgent revascularization [18]. Other studies suggest that XOIs may reduce mortality and cardiac events among individuals with hyperuricemia or gout [20–23], but this has not been evaluated in a large randomized trial. Therefore, delineating the underlying mechanism(s) of potential gout treatment benefit remains of pressing importance.

The Canakinumab Anti-Inflammatory Thrombosis Outcomes Study (CANTOS) demonstrated that reducing inflammation can reduce risk of cardiovascular disease [24], and it is possible that colchicine may reduce cardiovascular risk via a reduction in chronic inflammation. Alternatively, we recently reported that, compared to non-gout healthy controls, patients with gout have impairment of both brachial artery flow-mediated dilation (FMD) and nitrate-mediated dilation (NMD), measurements of endothelium-dependent and endothelium-independent (smooth muscle) arterial responsiveness respectively [25]. Impaired endothelial function assessed by FMD correlates with risk for future coronary events [26–29], and studies both in vitro and in animal models indicate that urate may impede the ability of endothelial cells to generate nitric oxide and thus limit arterial responsiveness [30, 31]. Therefore, both endothelial function and inflammation may be targets for lowering cardiovascular risk in patients with gout. The objective of this observational study was to determine the effects of initiating guideline-concordant gout treatment with colchicine plus an XOI (colchicine+XOI) on arterial endothelial function and systemic inflammation in patients with intercritical gout.

## Methods

### Study design and setting

This prospective observational study evaluated patients with intercritical gout who were being initiated on ACR guideline treatment to achieve target sU concentration by their physicians (rheumatologists or primary care physicians) in the ambulatory care offices of our three hospitals (NYU Langone Medical Center, Bellevue Hospital Center, and the VA New York Harbor Hospital). The study was approved by the institutional review boards of all three institutions, and all subjects provided informed consent.

### Participating subjects

Patients were eligible if they were at least 18 years of age and met ACR/EULAR classification criteria for gout [32], as well as ACR guidelines for initiating ULT [12], but were not currently receiving gout treatment. Patients were excluded if they had received colchicine or any specific ULT (allopurinol, febuxostat, probenecid, or pegloticase) within the past 30 days, or had prior intolerance or contraindication to treatment with colchicine, or with both allopurinol and febuxostat. Patients were further excluded if they were actively taking glucocorticoids or non-steroidal anti-inflammatory drugs on a chronic basis for any reason or had chronic kidney disease ≥ stage 4.

### Treatment protocol

Initiation of gout therapy was at the discretion of the subject’s physician, as was the choice and management of ULT. However, all subjects’ physicians agreed to follow a general plan provided by the investigators. Subjects slated for initiation of treatment underwent a baseline assessment of demographics, co-morbidities, and laboratory data including sU concentration, brachial artery function (FMD and NMD), and inflammatory markers (visit one). Treatment was initiated with colchicine 0.6 mg twice daily, with adjustment to once daily for intolerance or renal function (discretion of the treating physician). After 6 weeks of colchicine-only treatment, subjects underwent reassessment of sU concentration, brachial artery function, and inflammatory markers (visit two). While continuing colchicine, subjects were then started on XOI therapy (allopurinol 100 mg or febuxostat 40 mg daily at the discretion of the treating physician). sU was assessed every 3 to 5 weeks, and XOI was titrated to achieve a target sU concentration of ≤ 6.0 mg/dL (≤ 5.0 mg/dL in subjects with tophi). Once target sU concentration was achieved, subjects were maintained on stable doses of colchicine and XOI for four additional weeks, followed by reassessment of sU concentration, brachial artery function, and inflammatory markers (visit three). If any patient experienced a gout flare during a scheduled treatment assessment, the assessment was delayed until 1 week after resolution of the flare. The treatment approach was consistent with ACR 2012 Treatment Guidelines, which recommend the initiation of a prophylactic anti-inflammatory drug prior to or concurrently with an XOI, followed by titration of the XOI to target sU concentration [12, 15].

### Arterial function

Assessment of arterial function by FMD and NMD has been previously described [25, 33, 34]. Briefly, FMD was measured using a high-resolution duplex ultrasound imaging system (SonoSite, Inc., Bothell, WA, USA) connected to an 11-MHz linear array transducer with an axial resolution of < 0.1 mm and a computer-assisted edge detection system (AMS software). Brachial artery diameter (trailing edge of anterior intima-lumen interface to leading edge of posterior lumen-intima interface) was measured at end-diastole before and after transient arterial occlusion (forearm blood pressure cuff inflation to 50 mmHg above systolic blood pressure for 5 min) [35]. FMD was determined as percent change in brachial diameter from baseline to 1 min after occlusion release (mean of 3 separate measurements). Immediately after, NMD was determined as percent change in brachial artery diameter from baseline (pre-blood pressure cuff inflation) to 5 min after sublingual administration of nitroglycerin (0.4 mg). Whenever possible, assessments were performed in the morning in a fasting state, and subjects delayed any usual beta and/or calcium channel blocker doses until after the procedure. Subjects were permitted to opt out of NMD testing without being removed from the study and were excluded from NMD testing if their primary care physician or cardiologist recommended against it for any reason. In our laboratory, brachial artery reactivity in healthy adult subjects is 5.89% ± 2.88% (95% confidence interval = 4.53–7.23%), and brachial artery dilation response to nitroglycerin in adults without known heart disease is 24.7 ± 6.9% (95% confidence intervals 11.8%, 34.5%) [34, 36].

### Inflammatory markers

sU, high-sensitivity C-reactive protein (hsCRP), and erythrocyte sedimentation rate (ESR) were measured in standard clinical laboratories. For the measurement of IL-1β, IL-6, and myeloperoxidase (MPO), heparin-anticoagulated blood was centrifuged within 30 min of collection at 3000*g* for 10 min, and plasma aliquots were stored at − 80 °C until analysis. Plasma interleukin-1β (IL-1β), interleukin-6 (IL-6), and myeloperoxidase (MPO) concentrations were assessed using multiplex assays (Millipore Sigma, Burlington, MA) in duplicate on the MAGPIX multiplex instrument (Luminex Corporation, Austin, TX). Good clinical practices were followed to certify proper storage and daily and long-term quality control of reagents, instruments, and technique.

### Endpoints

The primary endpoint was the difference in FMD between visit one and visit three (colchicine+XOI). Secondary endpoints included differences in hsCRP, ESR, IL-1β, IL-6, and MPO concentrations, as well as NMD, between visit one and visit three. Additional secondary endpoints included differences in all parameters between visit one and visit two (colchicine-only).

### Sample size

For our primary endpoint of FMD, we posited a clinically meaningful change to be a 33–50% increase from baseline to final (colchicine+XOI) FMD. In a prior study of hyperuricemic individuals without gout, subjects experienced an 81% increase in FMD after treatment with allopurinol [37]. We calculated that recruiting 30 subjects would give us 83% power to detect a 60% improvement from baseline FMD. We also estimated power for our secondary outcome of change in hs-CRP analysis. We anticipated up to a 60% decline in hs-CRP with colchicine use, based on a study by Nidorf et al., in which non-gout patients with elevated hs-CRP were given colchicine for 30 days [38]. We estimated that we would have 80% power to detect a 30% change in CRP from baseline to endpoint with 30 subjects per group using a general linear mixed model. Power for all outcomes was estimated by Monte Carlo simulation using Stata v12.1 (College Station, TX). We recruited 38 subjects to increase our power and to allow for possible withdrawals/missing data points.

### Statistical analyses

Study data were collected and managed using REDCap (Research Electronic Data Capture) [39]. The demographics and clinical characteristics of subjects were summarized using descriptive statistics as mean ± standard deviation (SD) (normal distribution) or median [interquartile range] (skewed distribution) for continuous variables, or percentage (counts) for categorical variables. Endpoints were also expressed as mean ± SD or median [interquartile range]. For comparisons of repeated measures among baseline, colchicine, and colchicine+XOI treatments, analysis of variance (ANOVA) and related-samples Friedman’s two-way ANOVA by ranks test were used for normally distributed and skewed endpoints, respectively. For pairwise comparisons of repeated measures between baseline and after intervention, paired *t* test or related-samples Wilcoxon signed-rank test were used for normally distributed and skewed endpoints, respectively. Statistical significance of a *p* value < 0.05 was considered to be relevant. Statistical analyses were conducted in R-statistical package (www.r-project.org) and/or the IBM SPSS Statistics software, version 23 (IBM Corporation, Armonk, New York). Graphs were generated, and Pearson’s correlation coefficient for Fig. 2b was determined, using DeltaGraph 7.1.3, and is presented as mean ± standard error of the mean, or median [interquartile range].

## Results

### Baseline characteristics

Thirty-eight subjects with gout were enrolled in the study, of whom five (13.2%) were identified on physical examination as having tophi, and nine (23.7%) as having radiographic erosions on prior clinical X-rays. At study entry, the median time since receiving a gout diagnosis was 2 years (interquartile range, 0.5–9 years). Twelve subjects (31.6%) had previously received ULT (11 allopurinol and 1 febuxostat) (median time since prior ULT = 24 months; interquartile range, 4.5–48 months). Table 1 summarizes the baseline characteristics. All subjects were male. The mean age was 58.6 ± 13.1 years, and mean body mass index was 30.5 ± 4.4 kg/m^2^. Consistent with prior literature [5, 6], many of the subjects had cardiovascular, renal, and/or metabolic comorbidities and more than a quarter were current smokers.

**Table 1:** Baseline characteristics

Characteristic*	**Subjects (*n*** **= 38)**
Age (years)	58.6 ± 13.1
Body mass index (kg/m^2^)	30.5 ± 4.4
Years with gout at baseline (median [interquartile range])	2.0 [0.5–9.0]
Months since most recent gout attack (median [interquartile range])	1 [0.5–4]
Male (%)	100
Race (%)	
White	39.4
African American	47.4
Asian	13.2
Ethnicity (%)	
Non-Hispanic	86.9
Hispanic	13.1
Co-morbidities (%)	
Chronic kidney disease	18.4
Hypertension	68.4
Hyperlipidemia	55.3
Coronary artery disease	21.1
Diabetes mellitus	10.5
Smoking (%)	
Never	31.6
Previous	39.5
Current	28.9
Bony erosion (%)	23.7
Tophus (%)	13.2
Creatinine (mg/dL)	1.13 ± 0.28

*Unless otherwise noted, values are expressed as mean ± standard deviation or %

Thirty-two subjects (84.2%) were started on allopurinol and two (5.2%) were started on febuxostat. Four subjects were concurrently enrolled in another study (CSP #594: Comparative Effectiveness in Gout: Allopurinol vs. Febuxostat; ClinicalTrials.gov identifier NCT02579096) and received either allopurinol or febuxostat in a blinded manner. (Because these subjects received colchicine and an XOI together at the start of the study, they were not available for second visit (colchicine-only) analysis.) Of the subjects started on allopurinol, two were subsequently switched to febuxostat by their physicians, one for a possible hypersensitivity reaction (skin rash) and one because of raised liver enzymes.

### Follow-up

Thirty-two subjects completed the third visit; five subjects were lost to follow-up or failed to comply with treatment, and a single subject experienced arm discomfort after brachial artery function assessment and withdrew from the study. Twenty-nine subjects participated in NMD assessment at visit one, 25 at visit two, and 23 at visit three. Subjects who did not participate in NMD assessment at initiation did so because of personal preference (*n* = 3), medical contraindication to nitroglycerin (systolic BP < 110 mmHg or asymptomatic bradycardia at time of assessment) (*n* = 2), or lack of nitroglycerin availability (*n* = 4). One subject experienced transient hypotension after receiving nitroglycerin on visit one. He recovered fully, but NMD assessments were not performed at subsequent visits. One subject discontinued colchicine (but continued allopurinol) prior to visit three owing to the prohibitive cost of colchicine.

### Serum urate

Mean sU concentration at baseline was above the upper limit of normal for all subjects and met the standard physiologic definition of hyperuricemia of > 6.8 mg/dL (mean 9.12 ± 1.48 mg/dL). In overall comparisons, mean sU concentrations differed across the baseline, colchicine-only, and colchicine+XOI assessments (*p* < 0.0001) (Table 2). In pairwise comparisons, treatment with colchicine-only for 6 weeks did not change the mean sU concentration (9.69 ± 1.50 mg/dL), but after completing colchicine+XOI treatment, all subjects completing the study achieved their pre-specified target sU concentration (all subjects, 5.08 ± 0.66 mg/dL; non-tophaceous, 5.40 ± 0.72 mg/dL; tophaceous, 4.50 ± 0.32 mg/dL), representing a significant decline in mean sU concentration from baseline (*p* < 0.001) (Table 2). The mean allopurinol and febuxostat doses at treatment target were 384 ± 131 mg and 53 ± 23 mg/day, respectively. The median [interquartile range] time from initiating ULT (visit 2) to achieving sU target (visit 3) was 21 [13–28] weeks for the overall group (for non-tophaceous subjects, 17 [12–27] weeks; for tophaceous patients, 29 [26–42] weeks).

**Table 2:** Overall changes in endpoints across treatment steps

**Outcome**	**Mean ± SD or median [interquartile range]*N*(missing %)**			
	**Pre-treatment at baseline (visit 1)**	**Colchicine (visit 2)**	**Colchicine + XOI (visit 3)**	*p*value*
sU (mg/dL)	9.12 ± 1.48	9.69 ± 1.50	5.08 ± 0.66	< 0.0001
	38 (0%)	31 (18.4%)	32 (15.8%)	
FMD (% dilation)	1.93 ± 3.29	2.33 ± 2.95	3.04 ± 2.36	0.40
	38 (0%)	36 (5.3%)	31 (18.4%)	
NMD (% dilation)	17.45 ± 9.55	17.98 ± 6.07	17.25 ± 6.17	0.82
	29 (23.7%)	25 (34.2%)	23 (39.5%)	
hsCRP (mg/L)	4.28 ± 0.46	3.9 ± 0.53	3.0 ± 0.34	0.12
	36 (5.3%)s	32 (15.8%)	32 (15.8%)	
ESR (mm/H)	14.85 ± 8.72	12.90 ± 11.84	10.81 ± 6.59	0.03
	36 (5.3%)	29 (23.7%)	31 (18.4%)	
IL-1β (pg/mL)	2.60 [1.99–3.14]	2.64 [1.64–3.42]	2.05 [1.35–2.82]	0.01**
	34 (11%)	32 (16%)	28 (26%)	
IL-6 (pg/mL)	5.23 [3.84–24.74]	6.00 [2.65–32.66]	4.69 [2.22–14.90]	0.02**
	34 (11%)	32 (16%)	28 (26%)	
MPO (ng/mL)	160 [63–258]	150 [83–317]	182 [69–278]	0.87**
	34 (11%)	32 (16%)	28 (26%)	

*sU* serum urate, *FMD* flow-mediated dilation, *NMD* nitrate-mediated dilation, *hsCRP* high-sensitivity C-reactive protein, *ESR* erythrocyte sedimentation rate, *IL-1β* interleukin-1β, *IL-6* interleukin 6, *MPO* myeloperoxidase

*p values by univariate mixed-effect model ANOVA except where double asterisk (**) indicates related-samples Friedman’s two-way ANOVA by ranks test

### FMD and NMD

**Fig. 1:**
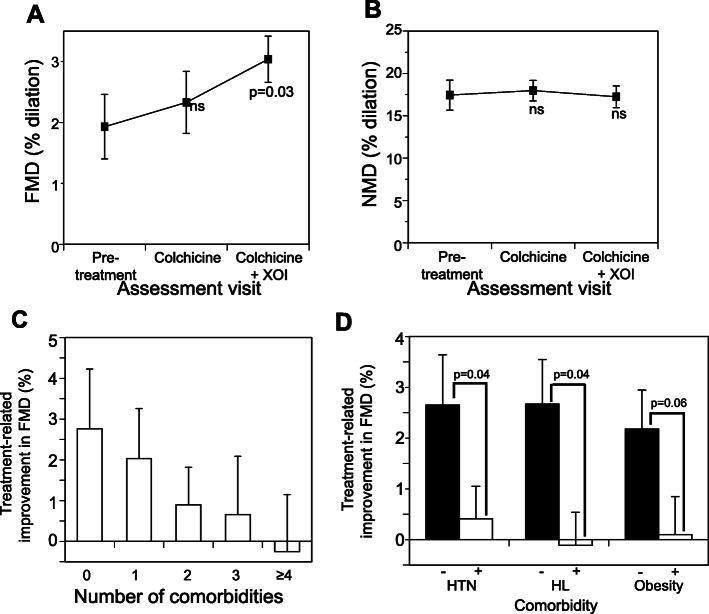
Impact of colchicine alone and colchicine+XOI on endothelial-dependent and endothelial-independent arterial responsiveness. **a** Flow-mediated dilation (FMD). **b** Nitrate-mediated dilation (NMD). **c** Relationship between number of co-morbidities and FMD improvement in response to colchicine+XOI. **d** Relationship between presence and absence of specific co-morbidities and FMD responsiveness to colchicine+XOI. HTN, hypertension; HLD, hyperlipidemia. Data shown are mean ± SEM

Mean FMD at baseline was 1.93% ± 3.29, numerically lower than that reported in non-gout control populations [25, 36, 40, 41]. Only 16% of subjects had a baseline FMD value greater than our laboratory norm for healthy controls. In overall comparisons FMD did not differ across the baseline, colchicine-only, and colchicine+XOI assessments (*p* = 0.40) (Table 2). In pairwise comparisons, treatment with colchicine-only for 6 weeks numerically improved the mean FMD (2.33% ± 2.95) from baseline (*p* = 0.72) (relative increase 20.7% from baseline), and after completing colchicine+XOI treatment, there was a significantly higher mean FMD (3.04% ± 2.36) with a 58% relative increase from baseline (*p* = 0.03) (Fig. 1a).

Mean NMD at baseline was 17.45% ± 9.55, numerically lower than that reported in non-gout control populations [34, 40]. Only 27% of subjects had a baseline NMD value greater than our laboratory norm for healthy controls. In contrast to FMD, NMD showed no improvement after treatment overall, or in pairwise comparisons (Table 2, Fig. 1b). Pairwise comparisons between colchicine-only and colchicine+XOI assessments were not significant for either FMD (*p* = 0.12) or NMD (*p* = 0.20).

### Impact of co-morbidities on FMD response

Overall, there was an inverse relationship between the total number of cardiovascular co-morbidities (diabetes, hypertension, hyperlipidemia, coronary artery disease, chronic kidney disease, and obesity) and the improvement of FMD in response to colchicine+XOI, such that patients with fewer comorbidities were more likely to experience FMD improvement (Fig. 1c). To further examine the role of comorbidities, we compared FMD results in patients with versus without the most common comorbidities in the population. Subjects without either hypertension or hyperlipidemia experienced significant post-treatment improvements in FMD, compared to subjects with these individual comorbidities (Fig. 1d). Similarly, non-obese subjects (body mass index < 30 kg/m^2^) experienced greater numerical improvement in FMD compared with obese subjects (Fig. 1d). The numbers of subjects with diagnosed coronary artery disease, chronic kidney disease, or diabetes mellitus were too small to permit similar comparisons. In contrast to FMD, the presence/absence and number of comorbidities had no consistent impact on NMD response to colchicine+XOI (data not shown).

### Inflammatory markers

**Fig. 2:**
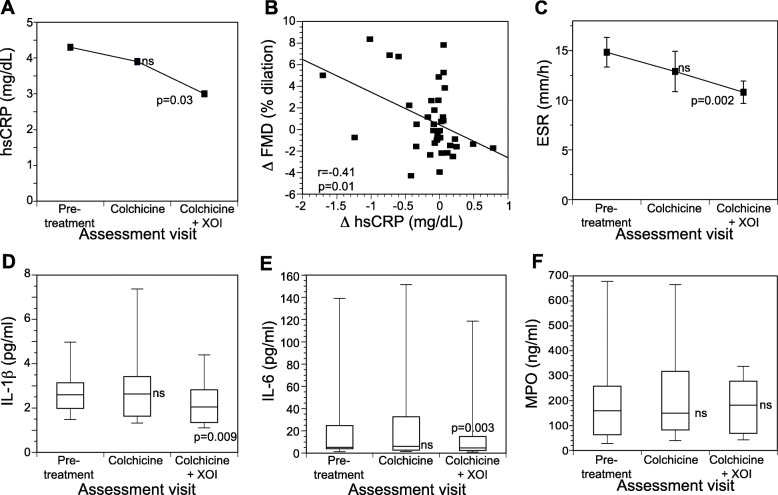
Impact of colchicine alone and colchicine plus a xanthine oxidase inhibitor (colchicine+XOI) on systemic inflammation in gout subjects. **a** High-sensitivity C-reactive protein (hsCRP). **b** Regression analysis showing relationship between changes in hsCRP and FMD in response to colchicine+XOI. **c** Erythrocyte sedimentation rate (ESR). **d** IL-1β. **e** IL-6. **f** Myeloperoxidase (MPO)*.* Data shown are mean ± SEM for **a**, **c**, and median/interquartile range for **d**–**f**

Mean hsCRP concentration at baseline was elevated above the clinical laboratory normal range (4.28 ± 0.46 mg/L). Overall, mean hsCRP concentrations did not differ across the baseline, colchicine-only, and colchicine+XOI assessments (*p* = 0.12) (Table 2). In pairwise comparison, treatment with colchicine for 6 weeks numerically lowered the mean hsCRP concentration (3.9 ± 0.53 mg/L) from baseline (8.9% relative reduction; *p* = 0.73) (Fig. 2a). After completing colchicine+XOI treatment, there was a significant further decline in mean hsCRP concentration (3.0 ± 0.34 mg/L) with a relative 30% reduction from baseline (*p* = 0.03) (Fig. 2a). Declines in hsCRP between the colchicine-only and colchicine+XOI assessments were borderline significant (*p* = 0.06). Improvement (decline) in hsCRP correlated moderately with improvement (increase) in FMD (R = − 0.41, *p* = 0.01) (Fig. 2b).

Mean ESR concentration at baseline (14.85 ± 8.72 mm/h) was not elevated above the upper limit of normal. Nonetheless, mean ESR concentration differed across the baseline, colchicine-only, and colchicine+XOI assessments (*p* = 0.03) (Table 2). In pairwise comparisons, treatment with colchicine-only for 6 weeks numerically lowered the mean ESR concentration (12.90 ± 11.84 mm/h) with a relative 13% reduction from baseline (*p* = 0.43) (Fig. 2c). After completing colchicine+XOI treatment, there was a significant decline in mean ESR concentration (10.81 ± 6.59 mm/h) with a relative 27% reduction from baseline (*p* = 0.002). Declines in ESR between the colchicine-only and colchicine+XOI assessments were borderline significant (*p* = 0.07) (Fig. 2c).

Median IL-1β and IL-6 concentrations, but not MPO concentration, differed across the baseline, colchicine-only, and colchicine+XOI assessments (Table 2). In pairwise comparisons, treatment with colchicine-only for 6 weeks did not change the median IL-1β (*p* = 0.91) or median IL-6 (*p* = 0.83) concentrations, but after completing colchicine+XOI treatment, there were significant decreases in both IL-1β (19.5% relative reduction; *p* = 0.009) and IL-6 (18.8% relative reduction; *p* = 0.003) concentrations compared with baseline (Fig. 2d,e). Declines in median IL-1β and IL-6 concentrations between the colchicine-only and colchicine+XOI assessments were significant (*p* = 0.02 for each analysis). Neither colchicine-only (*p* = 0.48) nor colchicine+XOI (*p* = 0.63) reduced median MPO concentrations compared with baseline (Fig. 2f).

In a sensitivity analysis, exclusion of the four patients enrolled in a parallel trial did not change the overall results or conclusions for either FMD/NMD or inflammatory markers (data not shown).

## Discussion

Our findings indicate that initiating guideline-concordant gout treatment with colchicine+XOI to target sU concentration results in significantly increased brachial artery flow-mediated vasodilation along with significant reductions in intercritical bloodstream hsCRP, ESR, IL-1β, and IL-6 concentrations, indicating that colchicine+XOI enhances vascular endothelial function and reduces baseline systemic inflammation in gout patients. Since endothelial dysfunction, elevated hsCRP, IL-1β, and IL-6 concentrations have all been associated with increased cardiovascular risk [26–28, 38, 42–44], and since lowering of hsCRP and IL-6 via anti-IL-1β strategies is associated with reduced rates of major adverse cardiovascular events [45, 46], these data suggest that gout treatment with colchicine+XOI may lower cardiovascular risk. Concentrations of MPO, a marker of neutrophil activation that is also associated with increased cardiovascular risk [47], were unaffected by colchicine+XOI treatment, indicating that not all aspects of inflammation may be modulated with initiating treatment in gout.

The ability of colchicine+XOI to improve endothelial function in our study is consistent with a prior study of individuals with asymptomatic hyperuricemia (hyperuricemia in the absence of gout), in which treatment with allopurinol 300 mg daily resulted in significant improvement in FMD [37]. However, the ability of XOI to improve endothelial function has not been previously reported in gout patients, the population most likely to receive these medications. Whether the benefit of xanthine oxidase inhibition on FMD is a consequence of sU lowering remains uncertain; some investigators have suggested that the vascular benefits of allopurinol may be due instead to its ability to reduce oxidant load [48]. Given that the current study was based on a treat-to-sU-target observational design in which all patients achieved a urate level ≤ 6.0 mg/dL, we cannot distinguish between the impact of sU lowering and any other effect of XOI treatment. Patients in our study also demonstrated a small albeit non-significant increase in FMD after colchicine alone, raising the possibility of a separate or additional colchicine effect.

Beyond improvement in FMD after colchicine+XOI treatment, we observed differences in the degree of FMD improvement based on the status of cardiovascular-associated comorbidities. Subjects with fewer cardiovascular comorbidities, and specifically, those without hypertension or hyperlipidemia, were more likely to experience FMD improvement in response to gout treatment than those with these conditions. These data suggest that established cardiovascular disease may limit the ability of the vasculature to respond to initiation of gout therapy and may be consistent with observations that urate lowering in hyperuricemic adolescents resulted in improvement in new-onset essential hypertension [49], whereas urate lowering in hyperuricemic adults with stage 3 chronic kidney disease was not similarly effective [50]. Current treatment guidelines recommend that chronic gout treatment be considered after multiple attacks, or after one attack in the setting of comorbidities. In this context, our data potentially argue for the importance of still-earlier initiation of gout management, prior to the establishment of comorbidities. Unfortunately, even under current, less-stringent guidelines, the quality of care remains poor in the general population of gout sufferers [51].

In contrast to FMD, we observed no improvement in NMD either after colchicine alone, or after colchicine+XOI treatment. Thus, although NMD was impaired, and although several studies suggest that urate may interfere with smooth muscle function [25, 52, 53], it does not appear that initiating gout treatment can reverse established smooth muscle impairment as measured by exogenous nitrate administration, at least after short-term treatment. The absence of treatment-related improvement in NMD may suggest that our FMD findings are attributable to increased bioavailability of endogenous nitric oxide, or other endothelium-derived vasodilator substances in vascular smooth muscle.

Whether the decrease in inflammatory markers that we observed after colchicine+XOI was due to extended exposure to colchicine, to the impact of XOI, or both cannot be determined based on the study design. In this context, it is interesting to note that Ives et al. have reported that xanthine oxidoreductase activity (the target of XOIs) can directly activate the NLRP3 inflammasome independent of urate generation, and that XOIs may therefore have urate-independent anti-inflammatory effects [54]. It was unexpected that initial treatment with colchicine alone did not result in significant reductions in inflammatory markers, since Nidorf et al. previously demonstrated that colchicine 0.5 mg twice daily significantly lowers hsCRP concentrations among non-gout patients with coronary artery disease after 4 weeks [38]. However, our study population was small, and we did observe non-significant decrements in some inflammatory markers after treatment with colchicine alone. It is possible that longer exposure, or assessment in a larger subject sample, would have revealed an independent effect of colchicine. It is also possible that gout patients are more resistant to the anti-inflammatory actions of colchicine compared to individuals without gout. Importantly, we observed an inverse correlation between hsCRP and FMD improvement, suggesting a link between inflammation and endothelial function. In contrast to 2012 guidelines, which specified a minimum of 6 months, 2020 ACR treatment guidelines suggest that prophylaxis be continued for a period of between 3 and 6 months [14, 15]. Whether ongoing use of colchicine, beyond the recommended prophylactic period, would have a durable cardiovascular benefit is a question raised, but not answered, by our study. Several other studies, though, do suggest a cardiovascular benefit of colchicine use in both the gout population and non-gout coronary artery disease populations [17, 18, 55]. Our studies also did not address the possible use of either NSAIDs (endorsed in both the 2012 and 2020 ACR gout treatment guidelines) or glucocorticoids (endorsed in the 2020 guidelines) as prophylaxis [14, 15]. We chose to study colchicine because of accumulating evidence supporting its cardiovascular benefit. In contrast, long-term use of both NSAIDs and glucocorticoids has been associated with increased cardiovascular risk [56]. However, such long-term risk does not preclude the possibility that initial prophylaxis with either of these two anti-inflammatory classes could have beneficial effect on FMD. Separate studies would be required to resolve this issue.

The strengths of this study include our use of a staggered treatment schedule, which allowed us to assess the initial effects of colchicine in isolation. Our observational design allowed treating physicians to select the XOI of their choice, reflecting real-world use and facilitating enrollment. Limitations of this study include our reliance on treating physicians to order laboratory studies, which led to cases of missing laboratory data. The fact that most patients received allopurinol was consistent with current US Food and Drug Administration recommendations [57], but prevented us from assessing the effect of febuxostat, a question of interest given recent reports that patients receiving febuxostat may have higher rates of cardiovascular and all-cause mortality compared with patients receiving allopurinol [58]. Moreover, our lack of an untreated control population may have limited our sensitivity to ascertain treatment-related differences. Our treat-to-target strategy was consistent with guideline-based management but resulted in a variable time period among patients between the second (colchicine-only) and third (colchicine+XOI) assessment. Our all-male subject population reflected the fact that we were recruiting for largely a male disease (gout) in a largely male hospital population (Veterans Affairs) but did not permit us to definitively extrapolate the effect of treatment on female gout patients. Even though a single technologist performed all FMD/NMD studies and the results were read by a blinded cardiologist, there likely remains considerable variability in FMD measurement. Our study population was limited, but establishes a context for larger, controlled studies to clarify these issues.

## Conclusions

In conclusion, initiating guidelines-concordant gout treatment with colchicine+XOI led to improved endothelial function, particularly among patients with fewer established cardiovascular co-morbidities. Colchicine+XOI also led to a decline in markers of systemic inflammation. Given that both impaired endothelial function and systemic inflammation are risk factors for adverse CV outcomes, these observations suggest that initiating guideline-concordant gout treatment may lower the risk for adverse CV outcomes through intravascular mechanisms.

## Data Availability

Data is available through the corresponding author.

## Abbreviations

ACR: American College of Rheumatology
EULAR: European League Against Rheumatism
ULT: Urate-lowering therapy
XOI: Xanthine-oxidase inhibitor
sU: Serum urate
FMD: Flow-mediated dilation
NMD: Nitrate-mediated dilation
hsCRP: High-sensitivity C-reactive protein
ESR: Erythrocyte sedimentation rate
IL-1β: Interleukin-1β
IL-6: Interleukin-6
MPO: Myeloperoxidase
SD: Standard deviation
SE: Standard error
ANOVA: Analysis of variance
